# The Stigma of Being HIV-Positive in Africa

**DOI:** 10.1371/journal.pmed.0020247

**Published:** 2005-07-19

**Authors:** William W Rankin, Sean Brennan, Ellen Schell, Jones Laviwa, Sally H Rankin

## Abstract

Rankin and colleagues argue that HIV-related stigma is fueling the epidemic, and disempowering women even further.

Jonathan Mann, founder of the World Health Organization's Global Program on AIDS and untiring advocate for justice for people with HIV/AIDS, addressed the United Nations General Assembly in 1987 [1]. His speech characterized the three major phases of an HIV/AIDS epidemic. After the initial silent spread of virus came the outbreak of ill health. The final stage, he said—the stage of social impact—is marked by stigma, grinding down its victims with shame and isolation.

Mann's tragically short life was devoted to protecting all who stood to be diminished by illness-related stigma and the erosion of elemental human rights [2]. Why were stigma and human rights so essential to the work of a medical doctor fighting an infectious disease?

## Fear of Stigma Fuels the HIV Epidemic

Stigma is of utmost concern because it is both the cause and effect of secrecy and denial, which are both catalysts for HIV transmission. Fear of stigma limits the efficacy of HIV-testing programs across sub-Saharan Africa, because in most villages everyone knows—sooner or later—who visits test sites [3,4]. While in some places the advent of free and accessible antiretroviral therapy has offered hope and encouraged people to go for testing, [5] stigma remains a barrier to testing even where treatment is available [6]. Without HIV testing, an essential first step to treatment, years may go by while people who are infected transmit the virus to others. When individuals finally become ill and seek care, treatment as a prevention strategy has lost much of its potential effectiveness.

Fear of stigma can cause pregnant women to avoid HIV testing, the first step in reducing mother-to-child transmission [7–9]. It may force mothers to expose babies to HIV infection through breast-feeding because the mothers do not want to arouse suspicion of their HIV status by using alternative feeding methods [10,11]. Fear of stigma, and the resulting denial, may even inhibit condom use in HIV discordant couples. Further evidence of how stigma leads to denial is the way in which newspaper obituaries avoid mentioning HIV/AIDS as a cause of death.

HIV-related stigma directly hurts people, who lose community support due to their real or supposed HIV infection. Individuals may be isolated within their family, hidden away from visitors, or made to eat alone [3]. These repercussions may or may not be simple acts of heartlessness. They may be a well-intentioned but ignorant attempt to preserve the family. In the community, the entire family may be sanctioned because one member is ill; in an impoverished society with no safety net of public services, this can be ominous for everyone [3].

In many African villages, an individual's, and a family's, life is closely intertwined with others. The same people have lived closely together for several generations, and there are few secrets. Inside families, caregivers may be largely concerned about contracting HIV through casual contact, and outside they fear the gossip that can greatly affect everyone's social standing. Neighbors and other customers, for instance, may refuse to purchase vegetables or poultry from someone associated with HIV [12]. In impoverished areas, this can devastate a family's chances of economic survival.

The language used to describe people living with HIV (such as “she is an HIV,” “he is a walking corpse”) clearly conveys stigmatizing attitudes. A particularly powerful example of stigmatizing language is found in parts of Tanzania, where an HIV-positive person is called *nyambizi*, or submarine, implying that the HIV-positive person is stealthy, menacing, and deadly [3,13].

People living with HIV can also experience a form of internalized stigma (Figure 1). Even without the burden of externally imposed social opprobrium, those living with a serious illness can face an enormous and painful inner struggle. They may eventually cease to be who they were, instead becoming a unitary “person with an illness” or—more damning—an “ill person,” a thing in which personhood and illness have completely fused. The philosopher Simone Weil characterized this assault of illness upon the self with the classical Greek notion of the soul—*Malheur* (affliction) stamps the soul to its very depths with scorn and disgust [14].

**Figure 1:**
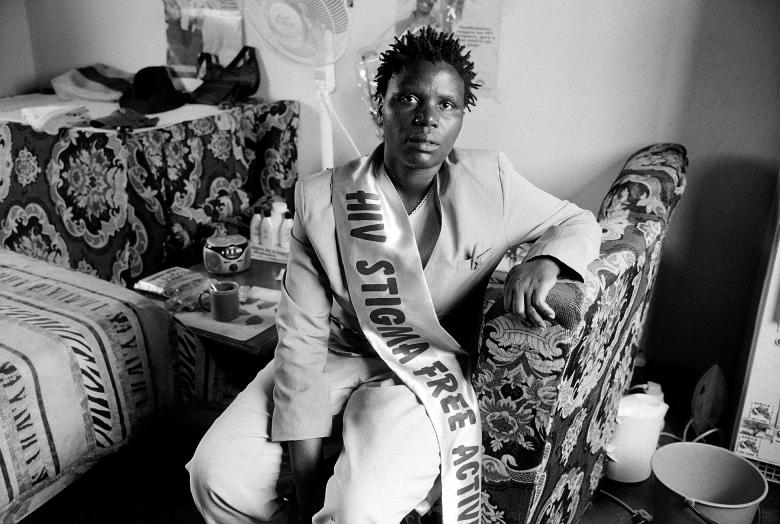
Kgalalelo Ntsepe, Who Was Named Miss HIV Stigma Free in 2003 In 2003, in Gaborone, Botswana, 14 women competed to become Miss HIV Stigma Free. The contest was won by Kgalalelo Ntsepe, who said: “It took a long time before I accepted my HIV status. At first, I almost wanted to kill myself. Eventually, I overcame my fears, even though my family and friends deserted me. But my church and my belief helped me to find a meaning in life again. I am Miss HIV Stigma Free. It's my responsibility to give strength to others. There's a life with HIV. There's life with AIDS.” (Photo: Copyright WORLD VISION/Sönke C. Weiss)

The combination of external stigma and internal oppression of the self may impose a heavy burden. In our experience of working with people with HIV in Africa, the result of this burden is often a downward spiral marked by fatalism, self-loathing, and isolation from others. And by shaming and silencing the very people who could credibly speak for HIV prevention and provide care for HIV-positive others, stigma fuels the HIV epidemic, consigning more people to suffering and death.

## Stigma in Society

Stigma is part of the attitudes and social structures that set people against each other. It impedes any countervailing forces for social equality. Certainly since Erving Goffman's seminal work on stigma in the early 1960s, stigma (plural stigmata) has been recognized as “an attribute that is significantly discrediting,” and it is known as a potent and painful force in individual lives [15]. Fueled by prejudice and appealing to it, stigma functions to diminish the person or group being targeted. Some commentators since Goffman have particularly examined stigma's broader social functioning. They have noted that while subordinating individuals or groups in society, the stigmatizing process also reinforces hierarchical patterns of privilege, where those at the top of a stratified society are pre-eminent over, and sometimes predatory upon, others at lower levels [16].

To see this perhaps more clearly, think of certain religious settings where punishment theories of illness causation are in force [17–19]. One such outlook presumes an aroused deity or ancestor bringing illness upon a person in retribution for an offense. This notion stigmatizes people struggling with their illness. It blames their sickness upon misbehaviors, while at the same time it rationalizes privileging the well over the ill. Punishment theories authorize communities to isolate or purge the “impure”—people whose illness or imagined “sinfulness” would contaminate the whole—while reassuring that virtue and social status will protect the righteous.

Clergy and other religious leaders are as susceptible as any to the temptation to exercise power over others. This imbalance of power is facilitated by such structured inequalities within churches as the preeminence of clergy over laity, of men over women, and even by the presumed superiority of the more “spiritual” over the less so. Under the influence of western missionaries, many African Christian organizations still promote evangelical formulae in which, it is taught, creation was originally good, but then the “fall” of humankind occurred, which is bad, and finally, redemption is available only for the chosen. This theological approach warrants valorizing or stigmatizing people as “saved” or “sinner,” “pure” or “impure,” “us” or “them,” and it strengthens the broader social stratifications within which stigma flourishes. What is weakened is the opportunity to apply healing insights from the rich Christian legacy of compassion, liberation, and hope [20].

## Gender and HIV

In much of sub-Saharan Africa, women are a subordinate group who are expected to become pregnant, bear children, and fulfill the sexual desires of their husbands without hesitation [20]. Such traditional assumptions, sometimes reinforced by the missionary religions, greatly benefit men while predisposing women to HIV infection. Often husbands carry HIV, while barrier methods of disease prevention, such as condoms, are proscribed, perhaps most vigorously by male-dominant religious organizations.

In addition to women's subordinate status in many societies, they are also frequently stigmatized as the vectors of HIV transmission, despite overwhelming evidence to the contrary. In Malawi, the term for a sexually transmitted disease, regardless of its origin, is “woman's disease.” [21] Husbands have beaten and/or abandoned wives thought to be HIV-positive, despite the fact that many women contract the virus from their husbands. Some women are subject to violence if they refuse a sexual overture, ask their husband to use a condom, or request an HIV test. If a husband should die, the wife's in-laws may seize her inheritance [22]. A woman exhibiting the independence needed to protect her health and self-esteem risks the disapprobation of her family and of the community.

Men are the clear winners of this arrangement in both social and economic terms, and many widows and their children, dispossessed or not, struggle against enormous odds simply to survive. Public attitudes, stigma among them, help to sustain the entire unjust system.

## Stigma and Human Rights

We marvel at the prescience and lucidity of Mann, a doctor who was dedicated to treating the whole person—both the physical ills and the emotional distress attendant upon these ills, including the stigma inherited from or imposed by societies where the oppression of some fortifies the privilege of others. Mann respected the healing potential of social justice in general, and of human rights in particular. He knew that a society in which multiple injustices routinely occur is itself not well, and he knew that widespread respect of human rights made less room for stigma and its harmful consequences.

Respect of human rights makes less room for stigma

The way to tackle social oppression of any kind is to introduce strategies that address underlying conditions of poverty, racism, and sexism that support such oppression [5]. This approach should be bolstered by sufficient legal and policy mechanisms to protect people subject to stigma and the erosion of human rights in general [5,23]. The same mechanisms should be functional and accessible to all.

To be effective, all HIV interventions should include an analysis of how stigma functions, how it enhances dominance and subordination in society, how it is that some win and others lose in the pernicious struggle for pre-eminence, and why it is that such a social scheme perversely flourishes in the first place [16].

Enlightened HIV prevention and care interventions (Figure 2) will empower the stigmatized through health education that lifts self-blame and shifts opprobrium to external, self-serving forces. While teaching respect for all through a more just society, these interventions will help people who are stigmatized to critique unjust societal dynamics and challenge assumptions and warrants of privilege.

**Figure 2:**
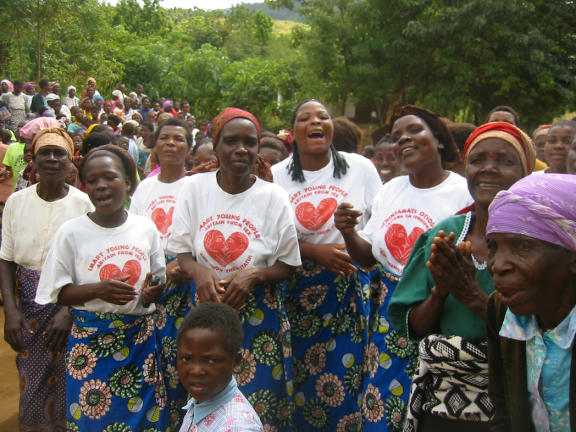
Village Caregivers This photograph shows some of the local women who work with Global AIDS Interfaith Alliance village-level projects in Africa, funded by the Bill and Melinda Gates Foundation. These women teach people about HIV/AIDS, help care for orphaned children, and visit and care for each person ill with AIDS every day. In doing so, they have helped to break down stigma in their villages. (Photo: Global AIDS Interfaith Alliance)

A tall order? Maybe, but Mann asked all of us—those struggling with illness and the presumably healthy—to make societies as healthy as their individual members.
